# Determination of Ochratoxin A in Wheat and Maize by Solid Bar Microextraction with Liquid Chromatography and Fluorescence Detection

**DOI:** 10.3390/toxins7083000

**Published:** 2015-08-05

**Authors:** Nabil Al-Hadithi, Philip Kössler, Petr Karlovsky

**Affiliations:** 1 Faculty of Pharmaceutical Sciences, Hashemite University, Zarqa 13115, Jordan; 2 Molecular Phytopathology and Mycotoxin Research, Georg-August-University Göttingen, Grisebachstrasse 6, Göttingen 37077, Germany; E-Mails: philip.koessler@uni-kassel.de (P.K.); pkarlov@gwdg.de (P.K.); 3 Department of Soil Biology and Plant Nutrition, University of Kassel, Nordbahnhofstrasse 1a, Witzenhausen 37213, Germany

**Keywords:** ochratoxin A, solid phase microextraction (SPME), solid bar microextraction (SBME)

## Abstract

Solid bar microextraction (SBME), followed by liquid chromatography with fluorescence detection (HPLC-FLD), for the quantification of ochratoxin A in wheat and maize was developed. Ground wheat and maize grains were extracted with acetonitrile-water-acetic acid (79:20:1, *v*/*v*/*v*), followed by defatting with cyclohexane, and subjected to SBME-LC-FLD analysis. SBME devices were constructed by packing 2 mg sorbent (C18) into porous polypropylene micro-tubes (2.5 cm length, 600 μm i.d., and 0.2 μm pore size). SBME devices were conditioned with methanol and placed into 5 mL stirred sample solutions for 70 min. After extraction, OTA was desorbed into 200 μL of methanol for 15 min, the solution was removed in vacuum, the residue was dissolved in 50 μL of methanol-water (1:1, *v*/*v*) and ochratoxin A content was determined by HPLC-FLD. Under optimized extraction conditions, the limit of detection of 0.9 μg·kg^−1^ and 2.5 μg·kg^−1^ and the precision of 3.4% and 5.0% over a concentration range of 1 to 100 μg·kg^−1^ in wheat and maize flour, respectively, were obtained.

## 1. Introduction

Mycotoxin ochratoxin A (OTA) is produced by numerous *Penicillium* and *Aspergillus* species such as *Penicillium verrucosum*, *Penicillium nordicum*, *Aspegillus ochraceus*, and *Aspergillus carbonariusm*, while new producers are continuously being discovered [1]. OTA occurs ubiquitously in plant products such as cereals, beans, groundnuts, raisins, coffee, beer and wine, as well as in certain animal products [2]. Cereals (wheat, barley, and oats) are the main source of human exposure to OTA [3]. Feedstuff is frequently contaminated with OTA, too; the levels vary with country and commodity, cereals being the most frequently-contaminated feed ingredient [4]. Interaction among OTA producers and other fungi suppresses or stimulates OTA production [5]. OTA has allegedly been implicated in a range of toxicological effects, including nephrotoxicity, mutagenicity, teratogenicity, neurotoxicity, and immunotoxicity, in animals and humans [6]. The mode of action appears to be associated with oxidative damage [7], though the effect OTA on epigenetic control has recently been demonstrated [8]. The widespread occurrence of OTA in food motivated continuous improvement of analytical methods for OTA during the last decade.

Liquid chromatography with fluorescence detection (LC-FLD) is the most used chromatographic technique for OTA determination [9,10,11,12,13]. Immunochemical methods [14] and thin-layer chromatography [15] offer high throughput due to the parallel analysis of many samples, while HPLC, with mass spectrometric detection, allows analyzing many mycotoxins simultaneously [16,17]. In spite of the advantages of alternative methods, LC-FLD remains the most popular method for OTA determination due to its high sensitivity and relatively inexpensive equipment available in most analytical laboratories. Sample pretreatment consisting of extraction, clean-up, and often preconcentration is required to remove matrix components and enhance sensitivity [18,19]. Common extraction methods are based on organic solvents such as acetonitrile, methanol, chloroform, and ethyl acetate, which are often acidified. The standard technique for clean-up and preconcentration of OTA is solid-phase extraction (SPE) [19]. A variety of SPE columns have been used including home-made columns filled with C8 sorbent [8], C18 column followed by cleanup on a mixed-mode polymer sorbent column [20], mixed-mode dispersive SPE [18], silica [11], molecularly-imprinted polymers [21], and immunoaffinity columns [9,13,22]. Liquid-liquid extraction (LLE) is also commonly used, often combined with SPE [9,11]. Interesting new developments include the use of ionic liquids as extraction solvents in LLE [18], cleanup of OTA by coacervation of reverse micelles [23], and liquid-liquid microextraction in porous hollow fibers [24]. Both SPE (except for the immunoaffinity columns) and LLE in a traditional setup require multistep protocols that are time-consuming and use large volumes of organic solvents; see [25] for a summary of the drawbacks. Immunoaffinity columns (IAC) offer unsurpassed specificity but are expensive, nor recyclable, have a limited binding capacity (heavily contaminated samples have to be diluted and submitted to a second cleanup, spending another IAC column), and a limited shelf life. The development of new simple, inexpensive, and environmentally friendly extraction and purification protocols therefore remains an important task in the analysis of OTA content in foodstuff.

Solvent-minimized solid phase microextraction (SPME) technique has been used for the determination of OTA in beer [26], coffee beans [27], cornflakes [28], and cheese [29]. SPME is a sorbent-based method in which extracting media is limited to μm-scale-sized sorbent, physically or chemically coated on fused-silica fiber [30]. A drawback of the technique is that SPME fibers are expensive and their lifetime is limited [25].

The recently developed solid bar microextraction (SBME) as an alternative miniaturized micro-solid phase extraction technique [31] that overcomes these difficulties by using only a few milligrams of a sorbent wrapped in a hollow fiber micro-tube [26,32,33]. Due to the porosity of the membrane, analytes are able to diffuse through and adsorb to the sorbent. Tumbling the solid “bar” in sample solution by stirring facilitates the extraction process. Porous membrane acts as a filter, excluding particles from the sample matrix from access to the sorbent. After the extraction, analytes are desorbed by immersing the device in a suitable organic solvent.

The most recent advancement in the determination of OTA was the use of aptamers. In spite of the promising results [34,35,36], the technique is not mature yet to be introduced into routine analytical practice. Immunochemical techniques for the determination of multiple mycotoxins in a single analysis were recently enhanced by the introduction of planar waveguides but the sensitivity remains a problem [37].

In this work, we investigated the application of SBME combined with HPLC-FLD to determine OTA content in wheat and maize grains. Parameters affecting the extraction efficiency such as sorbent properties, pH of the extract, extraction time, and composition of the desorption solvents were studied. Since cereal grains are the main source of OTA exposure [3], and maize is a staple grain in many countries, we have chosen wheat and maize as matrices for which the performance parameters of the method were determined.

## 2. Results and Discussion

### 2.1. Optimization of SBME Conditions

Acetonitrile-water extraction is the standard sample preparation for OTA determination in wheat and maize [13,16]. Elimination of acetonitrile before SBME is necessary; initial studies were therefore performed with the analyte dissolved in ultrapure water. The following SPME parameters were optimized for maximum OTA recovery: type of sorbent, number of SBME devices used, sample pH, extraction time, stirring speed, and desorption conditions. Concentrations of OTA of 4 to 5 μg L^−1^ were used in most optimization experiments in line with the maximum level allowed in raw cereals. Lower concentrations of 1 to 2 μg L^−1^ were used in experiments involving time course analysis to assure that the optimized time will be sufficient for lowest concentrations analyzed because the rate of adsorption/desorption is proportional to concentration. Optimization experiments were performed in triplicate. In line with IUPAC recommendation, extraction efficiency (recovery factor) was determined as the yield of OTA after extraction and apparent recovery was determined as the ratio of the peak area of a spiked sample, corrected for the background, to the peak area of the signal of pure standard [38].

**Figure 1:**
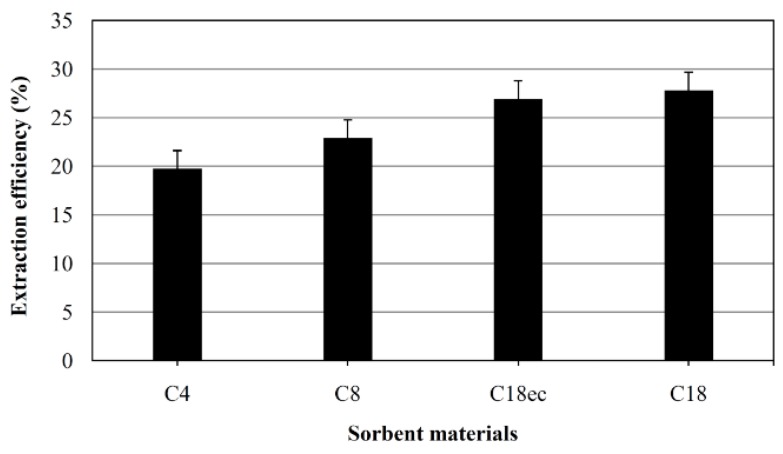
Effect of sorbent materials on SBME efficiency. Extraction conditions: 5 μg L^−1^ of OTA in 5 mL water, extraction time 90 min, stirring speed 500 rpm, desorption into 150 μL methanol in 10 min; three devices were used in each extraction. Error bars correspond to standard deviation.

The selection of an appropriate sorbent material is of major importance for the optimization of the SBME process. C4, C8, C18 and endcapped C18ec were compared in the extraction of OTA from 5 mL water samples. After extraction, the SBME devices were desorbed in methanol and the residue reconstituted in 50 μL of mobile phase and analyzed. As shown in Figure 1, C18 and C18ec showed the highest extraction efficiency; non-endcapped C18 was chosen as sorbent for further experiments.

**Figure 2:**
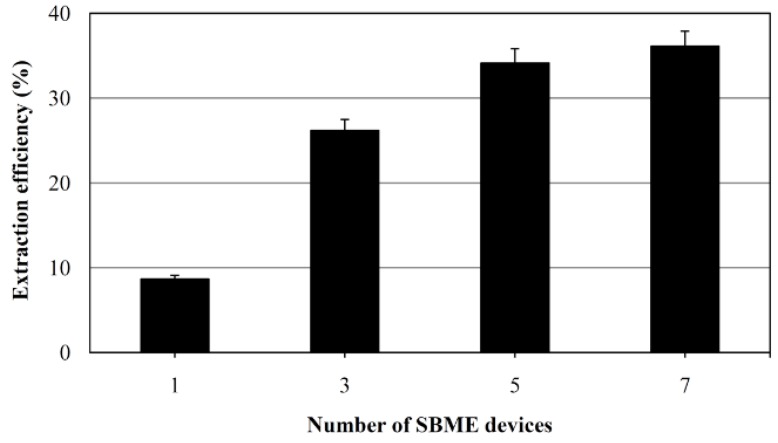
Effect of the number of SBME devices on extraction efficiency. Extraction conditions: 5 μg L^−1^ of OTA in 5 mL of 10 mM HCl, C18 sorbent, extraction time 70 min, stirring speed 300 rpm, desorption into 150 μL methanol in 15 min; three devices were used in each extraction. Error bars correspond to standard deviation.

The effect of using multiple SBME devices was evaluated by comparing extraction efficiency achieved with 1 to 7 devices for OTA solutions of 3 μg L^−1^ dissolved in 0.01 M HCl. As expected, extraction efficiency increased with the number of devices (Figure 2). However, when more than five devices were used, no additional enhancement was observed. Thus, five SBME devices were used for the remaining studies. Because OTA is a weak acid, samples for the extraction of OTA into organic solvents or for binding of OTA on hydrophobic matrices are usually acidified. pH of 2.0 was selected in our work.

**Figure 3:**
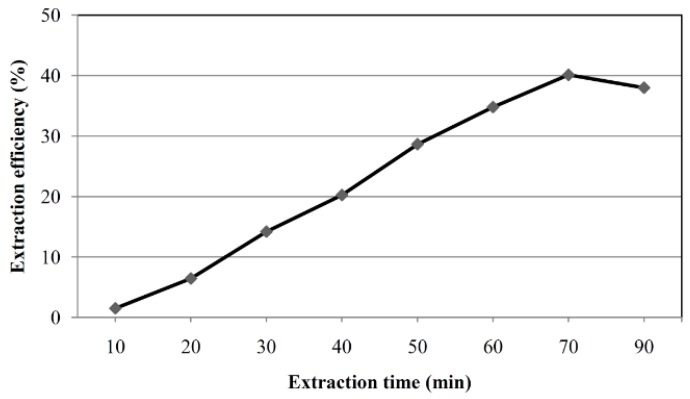
Kinetics of the OTA adsorption on SPME device. Conditions as in Figure 2, except for the concentration of OTA which was 1 μg L^−1^.

Adsorption of OTA on SBME sorbent is an equilibrium process. In order to determine the extraction time needed to achieve equilibrium, we extracted a solution of 1 μg L^−1^ of OTA in 0.01 M HCl for 10 to 90 min. As illustrated in Figure 3, the extraction reached equilibrium after 70 min. Thus, 70 min was selected for as the extraction time.

**Figure 4:**
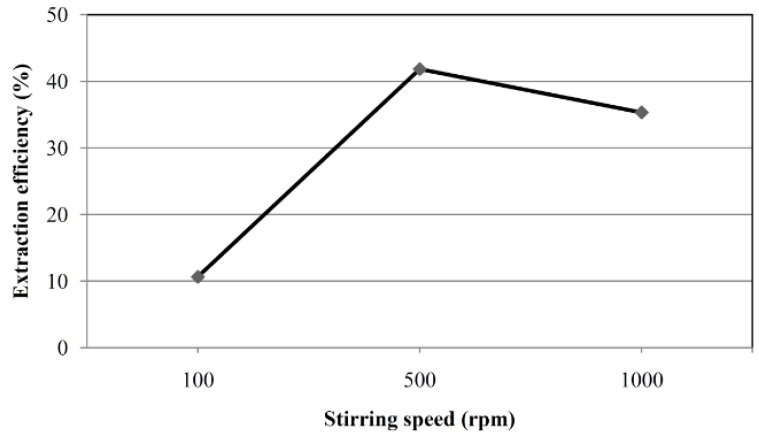
Influence of stirring speed on the recovery. OTA at a concentration of 4 μg L^−1^ was used, for other conditions refer to Figure 2.

Stirring reduces the time at which adsorption equilibrium is reached. Comparison of stirring rates of 100, 500, and 1000 rpm (Figure 4) showed that the highest adsorption was achieved at 500 rpm. Stirring at 1000 rpm caused formation of bubbles which tended to adhere to the surface of the fiber, impeding OTA transfer. Stirring at 500 rpm was chosen for further studies.

**Figure 5:**
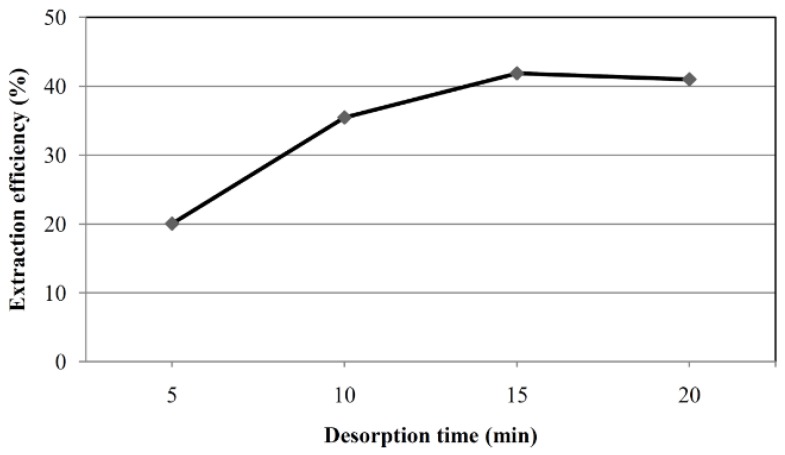
Effect of desorption time on the recovery of OTA in SBME. OTA at a concentration of 2 μg L^−1^ was used, for other conditions refer to Figure 2.

Selection of a suitable desorption conditions were also evaluated. Methanol, acetonitrile and acetone were used with sonication to release OTA from the sorbent. Methanol showed the best results (results not shown). Sonication time was also varied from 5 to 20 min. Figure 5 depicts the desorption profile of OTA (spiked at 2 μg L^−1^) showing that 15 min desorption time gave satisfactory results. No carryover of the analyte was observed when the SBME device after desorption was used again, which means that the devices are reusable. SBME devices were reused up to 20 times without compromising extraction efficiency (results not shown).

### 2.2. Analysis of Maize and Wheat Samples

Because wheat and maize grains are rich in fat which may compete with OTA for binding on SBME and compromise the performance of HPLC, acetonitrile-water extracts of ground grains were defatted with hexane (see Material and Method) prior to SPME cleanup.

**Figure 6:**
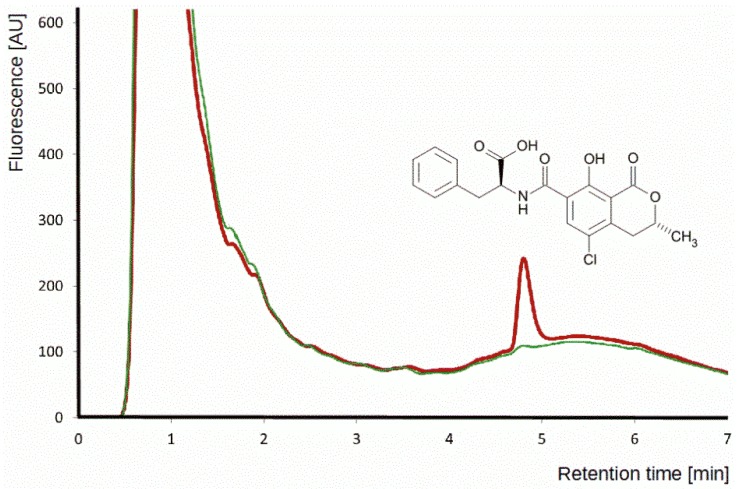
HPLC-FLD chromatogram obtained after SBME of wheat spiked with OTA at 10 ng g^−1^ (red) and wheat extract not containing detectable amounts of OTA (green). Fluorescence intensity in arbitrary units was plotted against retention time. The structure of OTA is shown above the peak of the mycotoxin at 4.9 min.

To quantify losses of OTA during sample pre-treatment, a procedure similar to one described by [27] was used. Two 5 g aliquots of ground, OTA-free wheat and maize grains were processed. The first aliquot was spiked with 2.5 μL of a 1 mg mL^−1^ OTA standard solution and left to equilibrate as described earlier (see “Sample Preparation”). Complete recovery would imply a final OTA concentration of 167 μg L^−1^ in 15 mL of acetonitrile-water-acetic acid extract. The second aliquot was processed as the first one, except that OTA was spiked into the final extract. Experiments were performed in triplicate. Peak areas of OTA signal of spiked sample, as compared to spiked extracts (apparent recoveries), were 97% ± 6% for wheat and 98% ± 4% for maize. The chromatographic conditions allowed a satisfactory separation of OTA from matrix components detectable with FLD at the same excitation/emission wavelengths in less than 6 min, as shown in a representative chromatogram in Figure 6.

The overall recoveries of OTA at a concentration of 5 μg·kg^−1^ were 35% and 38% for wheat and maize, respectively. At the spiking level of 50 μg·kg^−1^, recoveries of 36% and 39% for wheat and maize, respectively, were obtained. These values would be unacceptably low for standard SPE or LLE but they are common in non-exhaustive microextraction methods. For example, the recovery of OTA from beer by SPME under recommended conditions (60 min under stirring) was only 15% and the adsorption time would have to be extended to 10 h to reach equilibrium with a recovery of 60% [26]. Liquid-liquid-microextraction, too, suffers from long adsorption times and low recoveries [24]. The laboratory where SPME was invented has recently used the technique for the determination of OTA in cheese [29]; because their protocol is based on 50 mL loading solution, they had to increase the adsorption time to 8 h. In our protocol the time to equilibrium is much shorted (cf. Figure 3) because loading volume is 10-times smaller, but the low recovery is a problem that has to be addressed by future research. Even cleanup of OTA extracts based on standard-size SPE columns commonly lead to recoveries as low as 70%, as shown in a recent method comparison [10].

Performance parameters of the methods are listed in Table 1. LODs and LOQs were calculated with the Valoo software (Applica, Bremen, Germany) based on the standardization criteria DIN 32645 as defined by the German standardization committee [39]. LODs in the range of 0.92 and 2.48 μg·kg^−^^1^ are below the tolerance level for OTA in raw cereals permitted by EU directives (5.0 μg·kg^−1^) but the LOQ for maize is above this limit. The high sensitivity required for OTA analysis due to low regulatory limits poses a challenge to current analytical technology. A recently-published method for OTA in wine, which is an easier matrix than wheat and maize, generated comparable chromatograms for samples with the same level of OTA [18] (compare Figure 6 with Figure 4 in the cited publication), though a more sensitive laser-induced fluorescence detector was used.

**Table 1:** Performace parameters of SBME for OTA in wheat and maize grains.

Performance parameter	Wheat	Maize
LOD	2.5 μg·kg^−1^	0.9 μg·kg^−1^
LOQ	8.7 μg·kg^−1^	3.4 μg·kg^−1^
Correlation coefficient ( *r*) *	0.995	0.998
Repeatability as RSD ( *n* = 5)	5.0%	3.4%

* The coefficient of correlation was calculated for OTA at concentrations 5, 10, 25, 50 and 100 μg·kg^−1^.

Calibration curves for direct injection of OTA extracts without SBME enrichment were also constructed (data not shown); the LOD values found were about 100 times higher than the values reported in Table 1, indicating a large increase of sensitivity provided by SBME. Repeatability of the method was comparable or better than published methods based on liquid-liquid microextraction followed by HPLC-FD [18] and SPME followed by HPLC-MS/MS [29] but not as good as in a method that included double cleanup by chloroform partition and SPME followed by HPLC-FD [27].

## 3. Experimental Section

### 3.1. Chemicals and Reagents

Analytical standard of ochratoxin A (1 mg mL^−1^ in acetonitrile) was purchase from Fermentek (Jerusalem, Israel). Sorbent materials Chromabond C4 (C4), Chromabond C8 (C8), Chromabond C18 (C18), and Chromabond C18 endcapped (C18ec) were obtained from Macherey-Nagel (Düren, Germany). Hydrochloric acid (37%) and HPLC-grade organic solvents were obtained from Merck (Darmstadt, Germany). Q3/2 Accurel polypropylene hollow fiber membrane (600 μm i.d., 200 μm wall thickness, and 0.2 μm pore size) was purchased from Membrana (Wuppertal, Germany).

### 3.2. Sample Preparation

Wheat and maize grains were purchased in a local supermarket. Ground kernels (5 g) were spiked by adding the appropriate amounts of OTA in methanol to the flour. The samples were subsequently stored for three days at 40 °C to allow evaporation of the methanol and establish equilibrium between OTA and the matrix, simulating natural contamination. The samples were extracted with 15 mL of acetonitrile-water-acetic acid (79:20:1, *v*/*v*/*v*) for 12 hours on a shaker (100 rpm) at laboratory temperature and subsequently centrifuged at 1500 rpm for 10 min. The supernatants were defatted with 5 mL cyclohexane. Extraction solvent was dried in vacuum for 4 h at 45 °C. Dry residue was dissolved in 5 mL 0.01 M HCl and the solution was filtrated through 55-mm diameter GF/A glass microfiber filter (Whatman, Maidstone, UK). The solution was transferred into a glass vial of 10 mL and subjected to SBME.

### 3.3. Solid Bar Microextraction Procedure

The SBME device consists of sorbent materials enclosed within a hollow fiber polypropylene micro-tube (HF-PPMT). The SBME device was prepared as follows: HF-PPMT was manually cut with a sharp knife (scalpel blade) into pieces of 2.5 cm length. Each piece was closed from one side by means of a hot soldering tool, washed with methanol in an ultrasonic cleaner, and dried. A rod of stainless steel (0.4 mm diameter and 10 cm length) was used to introduce sorbent (~2 mg) through the open end into the lumen of the fiber. After filling with sorbent, the remaining open end of the tube was then heat-sealed to secure the contents. The SBME device was cleaned by sonication in methanol for 3 min and then stored in methanol until use.

A clean SBME device was placed in a 10 mL vial with sample extract (see sample preparation), and stirred at different speeds. The device tumbled freely in the sample during the extraction. After the extraction, the device was removed with a pair of tweezers, dried with lint-free tissue and placed in a 250 μL HPLC micro-vial containing 200 μL methanol. Desorption of OTA was facilitated by sonication. After removing the SPME device, the sample was dried in vacuum. The residue was reconstituted in 50 μL of methanol-water (1:1, *v*/*v*) and 20 μL were injected into HPLC-FLD unit for analysis.

### 3.4 Chromatography

The HPLC system consisted of a JASCO PU-2080 plus ternary pump, JASCO AS-2059-SF autosampler and a JASCO FP-2020 florescence detector (Jasco, Gotha, Germany). JASCO ChromPass chromatography data system (version 1.8.6.1; Jasco, Gotha, Germany) was used for data processing. Chromatographic separation was performed at 25 °C on a Kinetex*™* C18 column, 50 × 4.6 mm, 2.6 μm particle size, equipped with a C18, 4 × 3 mm pre-column (Phenomenex, Torrance, CA, USA). Eluents containing 7 mM acetic acid were acetonitrile-water (95:5, *v*/*v*, eluent A) or methanol (eluent B). The flow rate was 1 mL min^−1^. After an initial 0.2 min at 45% A, the proportion of B increased linearly to 98% within 10 min, followed by a washing step for 2 min at 98% B and 5 min equilibration at 45% A. The fluorescence detector was set up at excitation and emission wavelength of 333 and 455 nm, respectively. Injection volume of 20 μL was used throughout the study. Quantification was performed by integrating peak areas. Calibration curves for OTA were constructed in the range of 0.05–1.0 μg L^−1^.

## 4. Conclusions

SBME followed by LC-FLD was developed for the determination of OTA in wheat and maize samples. The method exhibits good precision and linear response over a wide concentration range. The apparent recovery was low (35%–39%) due to the non-exhaustive extraction technique used. Under optimal extraction conditions, LODs of 1.84 ng OTA on column for maize and 5.0 ng OTA on column for wheat were obtained, corresponding to 0.92 μg·kg^−^^1^ and 2.5 μg·kg^−^^1^ in maize and wheat, respectively. The method consumes a low amount of organic solvents, is easy to use, and cheaper than most currently-used cleanup procedures for OTA. Due to its low apparent recovery and high LOQ for wheat, the method is not suitable for official control of OTA level according to European legislative.
